# The synchronous occurrence of squamous cell carcinoma and gastrointestinal stromal tumor (GIST) at esophageal site

**DOI:** 10.1186/1477-7819-6-116

**Published:** 2008-11-05

**Authors:** Gian Paolo Spinelli, Evelina Miele, Federica Tomao, Luigi Rossi, Giulia Pasciuti, Angelo Zullo, Federica Zoratto, Jose Nunnari, Giovanni Codacci Pisanelli, Silverio Tomao

**Affiliations:** 1 Department of Experimental Medicine, University of Rome "Sapienza", Rome, Italy; 2 Universitary Oncology, S.M Goretti Hospital, Latina, Italy; 3 Department of Gynaecology Perinatology and Puericulture Science, University of Rome "Sapienza", Rome, Italy; 4 Gastroenterology and Digestive Endoscopy, "Nuovo Regina Margherita" Hospital, Rome, Italy; 5 Pathologic Anatomy, San Camillo-Forlanini Hospital, Rome, Italy

## Abstract

**Background:**

Esophageal squamous cell carcinoma is a relative common malignancy with a very poor prognosis, even adopting an integrated and multidisciplinary approach. According to the literature, gastrointestinal stromal tumors (GISTs) rarely originate from the esophagus. Moreover there are not reports of synchronous occurrence of squamous cell carcinoma and GIST at esophageal site.

**Case presentation:**

We describe a case of a 74 year old patient who underwent surgery for squamous cell carcinoma of the lower third of the esophagus with an incidental pathologic diagnosis of a concomitant GIST in the thoracic tract.

**Conclusion:**

In literature there is no evidence of concomitant squamous carcinoma and GIST of the thoracic esophagus, even if esophageal GISTs are sometimes described. The occasional finding of this neoplastic lesion underlines the importance of a carefully pathological diagnosis for its identification. Surgery, followed by a multidisciplinary approach remains the first-line treatment in both squamous and stromal neoplasm.

## Background

GISTs are the most frequent non epithelial neoplasms of the gastrointestinal tract, with a preferred gastric localization (about 60% in the stomach and 20–30% in the intestine). The esophageal location is very uncommon and represents approximately 5% of gastrointestinal GISTs. In addition, the majority of esophageal GISTs arise at the gastro-esophageal junction; therefore a GIST located at the level of the thoracic esophagus is extremely rare. Many esophageal GISTs are diagnosed after the onset of clinical symptoms, or sometimes discovered by chance, during routine examinations (diagnostic endoscopy procedures, transesophageal echocardiograms or surgical procedures carried out for other reasons). The diagnosed incidentally GISTs tend to be smaller in terms of centimetres and they already present the mutation at the KIT level. The elective treatment for these tumors is esophagectomy, paying particular attention to excision of the mucosa, sub mucosa and the muscular areas and of the surrounding periesophageal tissues.

## Case presentation

We describe a case of a 74 year old male patient, affected by a myeloproliferative syndrome. At the age of 26 he underwent a surgical gastrectomy for a hemorrhage caused by a perforated ulcer. Later, in December 2006, an upper gastrointestinal endoscopy revealed a neoplasm at the level of the thoracic esophagus. Multiple tissue biopsies histologically showed a scarcely differentiated squamous cell carcinoma. Subsequently all the necessary staging exams, including a total body Computed Tomography (CT) were performed. The CT did not show any suspected lymph nodes nor other lesions. In March 2007 the patient underwent a total esophagectomy and an esophago-gastroplasty with colon interposition for replacement of the esophagus.

### Histological examination

**Figure 1:**
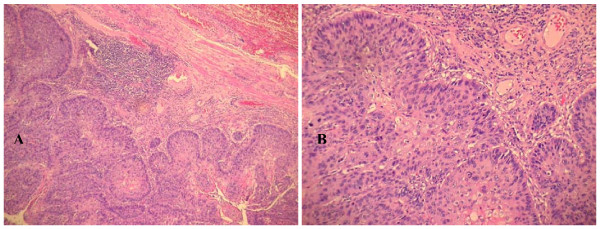
**Microscopic examination of squamous cell carcinoma**. A) Esophagus infiltrative squamous cell carcinoma (H and E 10×); B) Esophagus infiltrative squamous cell carcinoma (H & E 20×);

**Figure 2:**
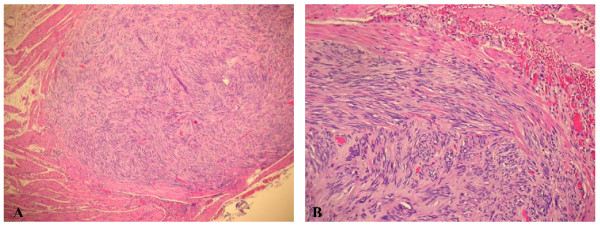
**Microscopic examination of GIST**. A) Intramural nodule of gastrointestinal stromal tumour (GIST) (H & E 10×); B) Fascicular arrangement of spindle cells with prominent nuclear palisade in GIST (H & E 10×);

The final histological findings confirmed the diagnosis of infiltrating squamous cell carcinoma; (Figure 1,A, B). The lesion extended into the sub mucosal layer, a grade G3 was diagnosed and the surgical margins were considered free of infiltration. Since the regional lymph nodes were not reported, the final staging was pT1 pNx. In addition, a sample was taken at a distance from the original tumor as an occasional finding in the esophageal muscular tunica. This showed a fused cellular intramural micro nodule with a maximum diameter of 0.2 cm, and with morphological characteristics suggesting a very low risk GIST (Figure 2A, B).

**Figure 3:**
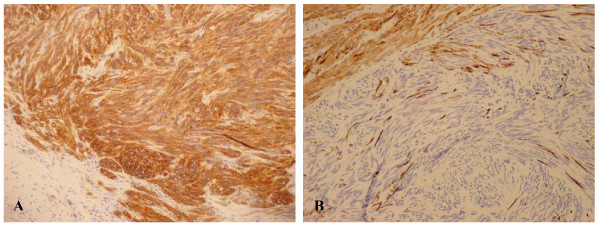
**Immuno – staining of GIST**. A) KIT (CD117) immuno-staining in GIST. The tumor cells show strong cytoplasmic and perinuclear positivity; B) Negative immuno-staining for Desmin in GIST.

The immunohistochemistry of the fused cells showed an intense and diffuse positivity for CD117, CD34 and CD99 and negative results for S100, desmin and actin (Figure 3A, B). The proliferate index Ki 67 was less than 5% with 4 mitoses per 50 HPF.

The aspects of this lesion reminded palisading-vacuolated spindle cell GISTs. In fact it showed nuclear palisading, resembling peripheral schwannomas and the typical feature of prominent perinuclear vacuolization.

In May 2007, a total body CT scan showed the presence of necrotic-hemorrhagic foci, of probable ischemic nature, in the cortical and sub cortical brain regions. At the same time, the thoracic region showed the presence of diffuse micro nodules in the lungs, associated with chronic bronchial and peribronchial wall thickening. We also noticed a modest splenomegaly (22 cm) and, at the aortic-pulmonary window, in the inferior pre-tracheal and right hilum areas, several lymph nodes of 13 mm. Considering the pathological and clinical staging, the patient did not require chemotherapy treatment, and would continue his follow-up with routine examinations.

## Discussion

The term Gastric Stromal Tumors was first coined in 1983 by Mazur and Clark [1], to describe a heterogeneous group of mesenchimal tumors that did not exhibit classic features of muscular differentiation. However only in 1998 Kindblom *et al *[2], revealed the expression of the antigen CD 117 and Hirota *et al*. [3], first identified a gain of function mutations at the level of the proto-oncogene c-KIT (CD 117) in this neoplasm, which is regarded to be pivotal in the development of most GISTs. These very suggestive neoplasias are extremely rare and tend to be localized at the gastric level (60–70%) and small intestine (20–30%); with smaller percentages these lesions have been described in other regions such as the large intestine, rectum, and omentum and with an incidence of less than 5% in the esophagus [4].

The median age of onset is 60–69 years and the symptoms are usually non-specific such as tiredness, abdominal discomfort and gastro-intestinal bleeding. The main instruments used for the diagnosis of GISTs are CT scans and PET scans.

^18^F-FDG PET seems to have lower sensitivity than CT for gastrointestinal stromal tumors staging. However PET is superior in monitoring therapeutic treatment response to imatinib in patients with malignant GISTs. CT and PET are complementary and PET/CT techniques have been shown to be useful in diagnosis, staging and treatment response in GISTs [5-7]. Based on parameters such as size and number of mitosis, these neoplasias can be subdivided into different risk classes: high, intermediate, low and very low.

Positivity for the CD117 is the key feature of GIST, but CD34 and nestin are other commonly expressed but less GIST-specific antigens. Moreover, these tumors can be positive for smooth muscle markers and generally negative for desmine. S-100 protein expression is rare and glial fibrillary acidic protein is not present. Keratine 18 and Keratine 8 are occasionally expressed.

CD99 is not required for GIST diagnosis, but as its immunoreactivity is not uncommon in a variety of soft tissue tumors, correlation of expression of this marker with that of other immunomarkers and with morphology is warranted [8].

Gastrointestinal (true) smooth muscle tumors, nerve sheath tumors, desmoids, inflammatory myofibroblastic tumors, inflammatory fibroid polyps, and undifferentiated sarcomas are the most commonly confused with GISTs. Rarely, poorly-differentiated carcinomas and histiocytic sarcoma can also take a part into differential diagnosis which is usually importantly aided by immonohistochemistry. These tumors have been reported as c-KIT negative but with other peculiar markers, generally not expressed in GISTs [9].

The differential diagnosis among GISTs and other epithelial neoplastic lesions of the gastro-intestinal tract is very important. In fact 95% of these tumors express the trans-membrane receptor with tyrosine kinase activity c-kit, and above all this group of lesions tends to show an interesting response (sometimes dramatic) to new target treatments [3,10-12].

Clinical studies have shown that the elective medical treatment for patients with inoperable lesions is imatinib (400 mg/die) with positive responses above 50% [13]. The use of other treatments such as Sunitinib, another tyrosine kinase inhibitor, has been approved in patients who do not respond to treatment with imatinib, and generally present a mutation of the exon 9 of c-kit [13,14].

Esophageal GISTs are rarely described in literature, and no cases of both GIST and squamous cell carcinoma have been reported. On the contrary, other kind of tumors have been found together (synchronous or metachronous) with GISTs in the gastrointestinal system, such as low grade malign lymphoma and GIST in stomach, colorectal cancer, gastric cancer, small bowel or mesenterium tumors and carcinoid of pancreas [15-18].

According to our epidemiological knowledge the hypothesis of a common etiopathogenesis at esophageal site, for GIST and esophageal squamous cell carcinoma cannot be supported. However we could hypothesize that development of these tumors may involve common carcinogen antigens, making the synchronous occurrence of GIST and other abdominal malignancy not only a coincidence.

## Conclusion

Our case report seems to be the only one described in a patient with a myeloproliferative syndrome, esophageal squamous cell carcinoma and GIST. The occasional finding of this latter neoplastic lesion underlines once more the importance of a carefully pathological diagnosis and a multidisciplinary approach. Of course, surgery [19,20] remains the first-line treatment in both squamous and stromal neoplasms.

## Consent

Written consent was obtained from the patient for publication of this case report.

## Competing interests

The authors declare that they have no competing interests.

## Authors' contributions

GPS conceived of the study, partecipated in its design and drafting. EM conceived of the study, partecipated in its design and drafting. FT participated in the design of the study and collect the clinical data. LR participated in the design of the study and collect the clinical data. GP participated in the design of the study and collect the clinical data. AZ participated in the design of the study and collect the clinical data. FZ participated in the design of the study and collect the clinical data. GCP participated in the design of the study and collect the clinical data. JN carried out the histopathological evaluation. ST conceived of the study, participated in its design and coordination and helped to draft the manuscript. All authors read and approved the final manuscript.
